# Gene and Metabolite Integration Analysis through Transcriptome and Metabolome Brings New Insight into Heat Stress Tolerance in Potato (*Solanum tuberosum* L.)

**DOI:** 10.3390/plants10010103

**Published:** 2021-01-06

**Authors:** Bailin Liu, Lingshuang Kong, Yu Zhang, Yuncheng Liao

**Affiliations:** State Key Laboratory of Crop Stress Biology for Arid Areas, College of Agronomy, Northwest A&F University, Yangling 712100, China; kongls@nwafu.edu.cn (L.K.); zhangyu3416@nwafu.edu.cn (Y.Z.)

**Keywords:** *Solanum tuberosum* L., gene expression, transcriptome analysis

## Abstract

Potatoes are particularly vulnerable to elevated temperatures, with short heat stress (6 h) inducing stomatal opening and reducing membrane stability and prolonged heat stress (3-day) decreasing the photosynthetic capacity of potato leaves. The integration of transcriptomics and metabolomics methods demonstrated that 448 heat upregulated and 918 heat downregulated genes and 325 and 219 compounds in the positive and negative ionization modes, respectively, were up- or downregulated in leaves in response to short and prolonged heat stress. Differentially expressed genes enriched in photosynthesis, cell wall degradation, heat response, RNA processing, and protein degradation were highly induced during heat exposure, and differentially expressed metabolites involved in amino acid biosynthesis and secondary metabolism were mostly induced during heat exposure, suggesting a possible role of these genes and metabolites in the heat tolerance of the potato. Metabolite and transcript abundances for the upregulation of flavone and flavonol biosynthesis under prolonged heat stress were closely correlated. Heat-induced gene expression in *Arabidopsis* *thaliana* shoots and potato leaves overlapped, and heat stress-responsive genes overlapped with drought stress-related genes in potato. The transient expression of four heat-induced genes in *Nicotiana benthamiana* exhibited increased heat tolerance. This study provides a new transcriptome and metabolic profile of the potato’s response to heat.

## 1. Introduction

Global warming has significantly affected the growth and yield of crops and thus represents a major threat to agricultural food production and food security. The extreme annual daily maximum temperature is predicted to increase by approximately 1 to 3 °C by the mid-twenty-first century [1]. Temperatures above the normal optimum are perceived by all living organisms as heat stress (HS), which has an impact on the development of different crop species. Heat stress disrupts cellular homeostasis and leads to severe changes in the structure, metabolic function, and physiological processes of plants [2]. Notably, heat stress is often accompanied by drought stress or other stresses that can cause extensive agricultural losses [3]. Therefore, it is urgent to understand the molecular thermotolerance mechanisms to breed and select heat-tolerant plant lines.

Considering the importance of the effect of global warming on crop yield, thermal stress in the potato is currently the research focus. Potato is one of the major staple crops in the world; however, it is a cool-season and highly heat-sensitive crop with an optimal growth temperature of approximately 20–25 °C and a tuber formation temperature of 15–25 °C [4]. Potato is the fourth most widely planted crop and a major food crop in China, where it is primarily grown in summer; however, the seasonal extreme high temperature in the main potato-producing area in the arid zones in northern China has a negative effect on potato growth and subsequent potato production. High-temperature stress is considered the main environmental factor affecting the growth, tuber yield, and quality of potato [5]. The tuberization signal is inhibited by heat exposure [6], and StSP6A, a tuberization signal, was shown to be decreased when temperatures increased. High temperature decreased the capacity of photosynthesis and starch accumulation because of the inhibition of carbon transport and CO_2_ fixation as well as the loss of chlorophyll content [6,7]. Elevated temperatures also have negative impacts on potato skin finish [8]. Additionally, high temperatures during tuber maturation have a negative effect on tuber dormancy and can cause potatoes to start sprouting [9]. Under the increasing temperatures caused by global climate warming, heat-tolerant potato varieties must be cultivated to sustain high yields under elevated temperatures [5].

To assess the effect of heat stress on potato growth performance, several research groups have identified differential expression patterns of genes in response to heat in different tissues via microarray analysis. The results from Rensink et al. [10] identified 416 heat-specific response genes using cDNA microarrays from potato roots and leaves, and 95 thermotolerance genes in potato were identified from cDNA libraries using a yeast-based functional screening method [11]. A subset of expressed genes was identified in the periderm exposed to high soil temperatures (33 °C) [12], and elevated temperature also had a significant effect on gene expression in leaves and tubers [6]. The results from Hastilestari’s study [13] showed that 671 differentially expressed transcripts were induced by a moderately increased temperature. These differentially expressed genes were involved in many biological processes and molecular functions as well as metabolite accumulation. Although the abovementioned studies have examined heat stress responses in potato, they have primarily focused on responses to mildly elevated temperature (30 °C) or immediate heat shock. Recently, extreme day temperatures above 35 °C and sustained high temperatures have occurred during the summer season. Compared to microarray analysis, RNA sequencing (RNA-Seq) is a high-throughput and low-cost sequencing method that can be used to investigate transcriptome changes at the genome-wide level; therefore, physiology, metabolism, and transcriptome are combined to analyze the transcriptomic and metabolic responses to short (6 h) and prolonged (3-day) heat exposure (35/33 °C, day/night) during potato plant development and identify putative regulators.

## 2. Results

### 2.1. Phenotypic Responses to Heat Stress in Potato

**Figure 1:**
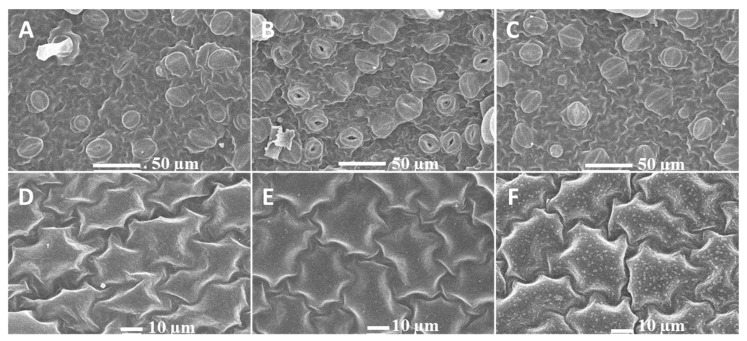
The characteristics of stomatal and leaf cuticle under heat stress in potato. (**A**,**D**) potato plants were incubated at 23 °C as control. B and E, potato plants were incubated at 35 °C for 6 h; C and F, potato plants were incubated at 35 °C for 3 days. (**A**–**C**), 500 magnification, scale bars = 50.0 µm. (**D**–**F**) 1000 magnification, scale bars = 10.0 µm.

**Figure 2:**
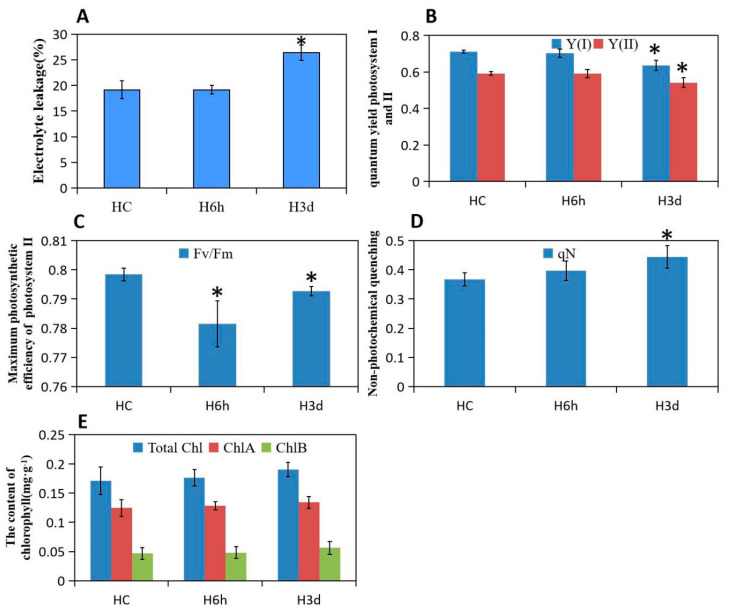
Membrane damage and photosynthetic activity after 6 h and 3 days incubation at 35 °C. (**A**). Electrolyte leakage assay. (**B**). Yield (I) and Yield (II) real-time quantum yield of PSI and PSII. (**C**). Fv/Fm maximum photosynthetic potential. (**D**). qN non-photochemical quenching. (**E**). The content of total chlorophyll, chlorophyll a and chlorophyll b. Data are represented as mean ± SE (*n* = 3), asterisks indicate significant differences between treatments as estimated by Student’s t test (*p* < 0.05).

Heat stress induces several alterations in plant growth, cell division, enzymes, hormones, and membranes. Plant cuticles outside of the epidermis of leaves have a protective effect against abiotic stress, and a large number of waxy crystals in the epidermis of potato leaves are formed after 3 days of heat treatment. We also found that most stomata are opened after 6 h of heat treatment, although this was not obvious for control and 3-day heat-treated leaves, indicating that potato leaves are responsive to short heat stress by regulating stomatal activity (Figure 1). Electrolyte leakage assays have been widely used to examine the level of plant tolerance to various stresses, including heat stress. The percentage of electrolyte leakage from 3-day heat-treated leaf tissue was significantly higher than that from control and 6-h heat-treated leaf tissue (Figure 2A).

High temperature seriously affects plant leaf photosynthetic performance. Compared to the control, we noted that prolonged heat stress caused a significant decrease in the Fv/Fm, yield (I), yield (II) and nonphotochemical quenching (qN) in potato; however, short heat exposure had no effect on these photosynthetic fluorescence parameters except for qN (Figure 2B–D), indicating that sustained high temperature over 6 h of exposure severely damages the PSI and PSII, the maximum photosynthetic efficiency, and the photoprotective capacity of potato leaves. However, the contents of photosynthetic pigments, including chlorophyll a, chlorophyll b, and total chlorophyll, were not significantly reduced after short and sustained heat exposure (Figure 2E).

### 2.2. Heat Stress Induced Transcriptional Changes in Potato Leaf

To understand the transcriptome changes in the potato leaf response to HS, totally expanded third leaves of cultivar “Hezuo 88” were isolated from 6-h and 3-day heat-treated (35/33 °C) plants. The same types of leaves taken from plants under normal growing temperature (23 °C) were used as controls. Illumina RNA sequencing experiments were conducted on total RNA from these tissues, and two biological replicates were performed. Approximately 45 million valid reads were generated from each of the six samples and aligned to the Genome Sequencing Consortium (PGSC) using STAR (v2.5.1b). Gene expression was quantified as FPKM values. We identified 18,052 genes to be expressed in leaves from the control, 6-h, and 3-day heat stress-treated samples. Differentially expressed genes were identified using the DESeq2 package (1.10.1) in R based on a negative binomial distribution, and a total of 448 heat upregulated genes and 918 heat downregulated genes were detected in the heat treatment group compared to the control group. By setting the fold change value $\left(\log_{2}FC\right)$ > 2 and FDR < 0.05 as cut-offs (Table S1), we identified 160 and 130 genes that were specifically upregulated and 538 and 94 genes that were specifically downregulated in response to short and prolonged heat stress, respectively. Among them, 35.3% of the upregulated genes (158 out of 448) and 31.2% of the downregulated genes (286 out of 918) were shared by the 6-h and 3-day heat-treated plants (Table 1). These commonly expressed genes might participate in both transient stress signaling sensing after the start of heat stress exposure and adaptive responses to sustained stress. Interestingly, the number of significantly up- and downregulated genes was reduced after 3 days of high-temperature exposure, indicating that potato plants adapt to tolerate sustained heat stress since the expression levels of several genes were similar to those in the untreated samples.

### 2.3. Functional Category Enrichment

To better understand the functional classification of short and prolonged heat-induced gene expression data, MapMan software was used to group the DEGs into functional groups or ‘bins’. As illustrated by the ‘metabolism overview’ (Figure S1), 2 major CHO metabolism genes (SuSy degradation, beta-amylase), one cell wall protein gene (AGP), and 3 cell wall degradation genes (beta-1,4-glucanases, pectate lyases, and polygalacturonases), 5 amino acid metabolism genes (glutamate decarboxylase, threonine, tryptophan, lysin, tyrosine), 2 lipid metabolism genes (steroids), 2 tetrapyrrole synthesis genes, and 11 secondary metabolism (isoprenoids, flavonoids, phenylpropanoids, simple phenols) were strongly induced at 6 h compared with the untreated samples, whereas only 2 secondary metabolism genes (isoprenoids, phenylpropanoids), one cell wall degradation gene (mannan-xylose-arabinose-fucose) and 2 cell wall modification genes, and one minor CHO metabolism gene (raffinose) were highly induced after 3 days of heat stress. In contrast, the negative impact of heat on metabolism became evident when many genes associated with the photosynthetic machinery (light reaction), major CHO metabolism (starch synthesis gene, sucrose degradation g), nucleotide metabolism (dUTP diphosphatase, thymidylate kinase, aspartate transcarbamoylase), lipid metabolism (phospholipid synthesis, fatty acid dehydrogenase, fatty acid synthesis, lipid degradation), and secondary metabolism (isoprenoids, phenylpropanoids) showed downregulation at 6 h of heat exposure, the repressive effect of prolonged heat exposure at 3 days was further expanded to a few genes associated with nucleotide metabolism, cell wall modification, and lipid metabolism. On the other hand, many genes shown in the MapMan display of metabolism overview were consistently up-or downregulated during heat treatment. The consistently increased expression of genes including 7 photosynthesis genes (light reaction, Calvin cycle), 8 secondary metabolism genes (alkaloid-like, phenylpropanoids), 7 cell wall degradation and modification genes (cellulose synthase, pectate lyases, and polygalacturonases), 3 lipid metabolism genes (steroids, lipases), 2 amino acid metabolism genes (aromatic AA, aspartate), one fermentation gene (ADH), one TCA gene (aconitase), and one redox gene (ascorbate). The consistently decreased expression of genes was mainly involved in the process of cell wall modification and secondary metabolism (simple phenols). The functional categories related to photosynthesis, cell wall degradation, and secondary metabolism were overrepresented among heat-responsive genes, supporting roles for those functional categories in the heat stress response in the potato.

Cellular processes associated with heat stress were shown by ‘cellular response overview’ function of MapMan. The heat stress response was the most abundant functional category during heat treatment. After 6 h of heat stress, 3 genes encoding HSP17 family proteins (HSP17.4, HSP17.6, HSP17.8), 2 genes encoding HSP22.0, one each for HSP18.2, HSP20-like chaperone, HSP26.5, and HSP81-1 were upregulated, whereas only 2 genes encoding for DnaJ domain containing proteins were downregulated. After 3 days of heat stress, only 2 genes involved in the HSP17.6A and DnaJ protein homolog were upregulated. Twenty-six heat stress response genes were consistently up- or downregulated during treatment, 24 genes that expression was consistently increased for 10 HSP17 genes (HSP17.4, HSP17.6, HSP17.8), 5 HSP70 genes (HSP70, HSP70b, HSC70-1, HSC70-7), 2 HSP80 genes (HSP80-1), one each for HSP15.7, HSP21, HSP22, HSP23.6-MITO, HSA32, DnaJ protein homolog, and BCL-2- associated athanogene 6 gene, only 2 genes encoding DnaJ heat shock N-terminal domain-containing protein (J11) and HSFA4A were consistently reduced, indicating that these gene families are strongly associated with sustained heat stress response in the potato.

**Figure 3:**
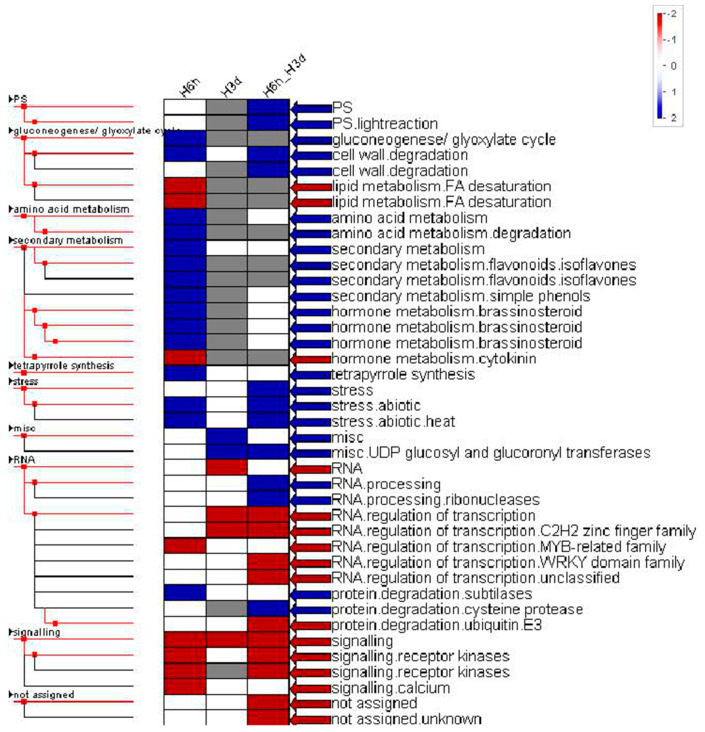
Enriched functional categories of induced gene expression in heat-stressed samples versus control samples. Each vertical column represents the genes that are dramatically up (blue) or down (red) regulated when comparing the H6h and H3d with HC respectively. Right arrows to color bar represent the detailed functional category derived from MapMan in the color bar ranging from −2.0 to +2.0. HC, control potato leaves; H6h, potato leaves after 6 h heat exposure; H3d, potato leaves after 3 days heat exposure.

The PageMan program was further used to enrich functional categories to identify heat-induced or reduced cellular processes. DEGs enriched in amino acid turnover, cell wall degradation, hormone metabolism (brassinosteroid), secondary metabolism (isoprenoids, simple phenols), heat stress, and protein degradation were highly induced after 6 h of heat exposure, while DEGs enriched in lipid metabolism (FA desaturation), hormone metabolism (cytokinin), RNA regulation, and signal transduction were mostly suppressed. DEGs enriched in the miscellaneous group (UDP glucosyl and glucoronyl transferases, glutathione S transferases, cytochrome P450, peroxidases, short chain dehydrogenase/reductase) were highly induced after 3 days of heat exposure, while DEGs enriched in RNA processing and regulation and signal transduction were mostly suppressed. DEGs enriched in photosynthesis, cell wall degradation, heat stress, RNA processing and protein degradation were highly induced and DEGs enriched in RNA processing and regulation and signal transduction were highly suppressed at both 6 h and 3 days of heat exposure (Figure 3). The responses at 6 h of heat exposure indicated that the heat was sensed and the defense response was induced, whereas after sustained heat stress for 3 days, an adaptive response to heat or inhibitor effects on potato growth were induced.

### 2.4. Transcriptional Regulation Associated with Short and Prolonged Heat Stress

The effects of heat on the expression of transcription factor genes were also examined. Table 1 shows that 70 transcription factors (TFs, 14 upregulated or 56 downregulated) were differentially (*p* > 0.05) expressed after short heat exposure and 21 TFs (7 upregulated or 14 downregulated) were differentially expressed after prolonged heat exposure. Among them, 36 TFs (6 upregulated or 30 downregulated) were overlapped under short and prolonged heat exposure (Table S2). These heat-responsive TF genes belonged to 18 different TF families, indicating diverse regulatory functions. Under short heat stress, one homeobox, G2-like, C2C2-GATA, C2C2-CO-like, Psudo ARR, and bHLH, 2 MYBs, 3 AP2/EREBPs TF genes were significantly upregulated; under prolonged heat stress, one each for PHD finger, MYB-related, bHLH, GRAS, AP2/EREBP, and Aux/IAA TF gene was significantly upregulated; and under both short and prolonged heat stress, one each for bHLH, MYB, and C2C2-DOF TF gene was significantly upregulated (Figure S2). Interestingly, 13 WRKY genes and 2 bZIP genes were identified to be downregulated after heat exposure. WRKY transcription factor was reported to performe a dual function of responding to heat and cool. AtWRKY25, AtWRKY23 and AtWRKY33 are the gene that response to heat tolerance by expressing heat inducible genes via AtHsps, AtMBF1 and AtZats [14]. The transcription factor bZIP60 was reported to modulate the heat shock response in maize [15]. Potato bZIP gene family has also been characterized in response to several abiotic stresses, including heat [16]. The other TF family members, such as PHOR1, ARR, C2H2 zinc finger family, and MYB, as regulators in the heat stress response is poorly understood, but providing partial evidence that these TFs may participate in the regulation of heat responsive process as negative or positive regulators.

### 2.5. Comparison of Heat Stress-Responsive in Potato with Heat Stress-Related Genes in Arabidopsis thaliana Shoots and Drought Stress-Related Genes in Potato

**Figure 4:**
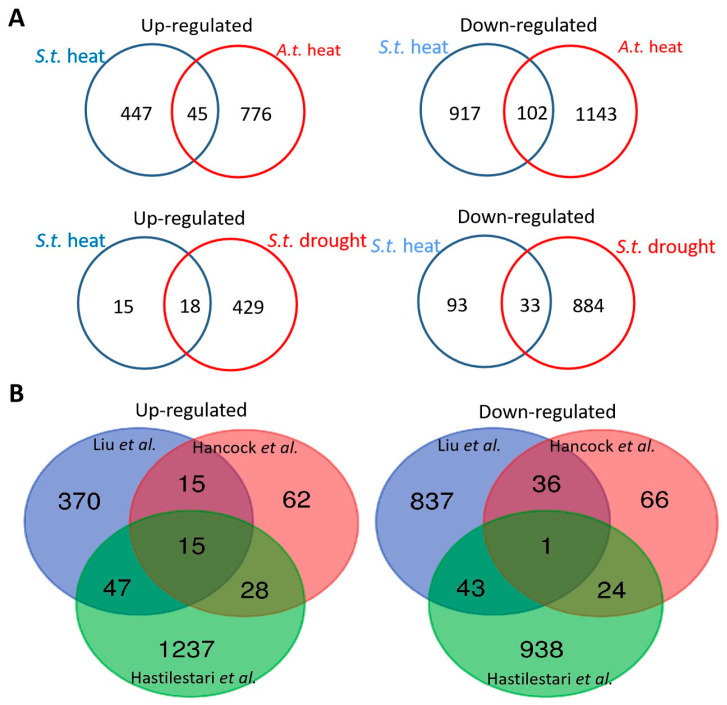
Number of common elements in the heat stress and published datasets. (**A**) Venn diagrams depict the overlap of induced and repressed genes in heat-stressed potato versus heat-stressed *Arabidopsis thaliana* (*A.t.*) shoots, as well as induced and repressed genes in heat-stressed potato versus drought-stressed potato. (**B**) Venn diagrams depict the overlap of differentially expressed genes in the heat stress and published genes as described by Hancock et al. (2014) [6] and Hastilestari et al. (2018) [13].

To investigate leaf-specific and conserved heat stress response gene expression between different species and plant organs, we compared the DEGs from the heat-treated potato leaves with the DEGs from heat-exposed *Arabidopsis thaliana* shoots [17]. Although the time point of heat treatment between the two experiments differed for potato and *Arabidopsis thaliana*, the number of DEGs in potato leaves was of a similar magnitude to that in *Arabidopsis thaliana* shoots (776 upregulated; 1143 downregulated). A comparison of the best BLAST hits of the DEGs in potato leaves with the DEGs in *Arabidopsis thaliana* shoots showed an overlap of 45 upregulated and 102 downregulated genes between potato leaves and *Arabidopsis thaliana* shoots (Figure 4A and Table S3).

To further identify whether heat-induced or repressed genes showed cross-responsiveness to other abiotic stresses, these heat response genes in our study were compared to previously released potato drought-responsive DEGs in leaves [18]. We identified an overlap of 33 heat-repressed and 18 heat-induced genes between heat- and drought-responsive DEGs (Figure 4A and Table S4). Among them, 15 induced genes were associated with heat shock protein, one gene was associated with peptidylprolyl isomerase, and 2 genes were conserved genes of unknown function.

In addition, two experiments were performed using microarrays to identify heat-responsive genes from potato leaves. A comparison of our data sets with that of Hancock et al. [6] and Hastilestari et al. [13] showed an overlap of 30 and 62 induced genes and 37 and 44 repressed genes, respectively (Figure 4B, Table S5). We noticed only a small overlap of 15 upregulated genes and 1 downregulated gene (fold change greater than 2) among the three studies, suggesting that these commonly induced genes were likely to take part in heat tolerance and/or heat response in potato leaves. Some of these genes are well-known members associated with the plant constitutive heat stress response, including small heat shock protein (HSP) genes and cytosolic ascorbate peroxidase genes. However, this overlapping group also includes other as of yet uncharacterized genes, such as BCL-2-associated athanogene 6 and nonspecific lipid-transfer protein. Further functional analysis showed that 15 DEGs were involved in photosystem II and the remaining genes were involved in lipid metabolism, heat stress, and misc (glutathione S transferases), indicating that they are potential candidates for maintaining the development of potato cultivars under elevated temperatures.

### 2.6. Transient Expression of Heat Induced Genes in Nicotiana benthamiana Leaves

To further characterize the function of heat tolerance genes, transient expression experiments of 3 HSP genes, HSP17.6-1, HSP17.6-2 and HSP17.7, and one cellular metabolism-related gene, StNLP2, were performed in *Nicotiana benthamiana*. After agroinfiltration-mediated transformation, the plants were incubated in a growth chamber at 45 °C under 12 h photoperiod. After 24 h of heat treatment, we found that agroinfiltration with StHSP17.6-1, StHSP17.6-2, StHSP17.7, and StNLP2 overexpression constructs expressing the HSP17.6-1, HSP17.6-2, HSP17.7, and NLP2 genes resulted in lower levels of cell membrane damage than the control (Figure S3).

### 2.7. RNA-seq Validation by RT–qPCR

We used RT-qPCR to examine the expression patterns of 20 randomly selected DEGs in potato leaves after 6 h and 3 days of heat exposure. The results indicated that 18 out of the 20 genes identified between the RT-qPCR and RNA-seq assay were consistent, whereas the 2 genes PGSC0003DMG400021877 and PGSC0003DMG400012436 showed transient increased expression after 6 h heat exposure but recovered to levels similar to the control level after 3 days of heat exposure via RT-qPCR while their expression continued decreasing after the start of heat exposure and under prolonged heat stress via RNA-seq (Figure S4). This result indicates that the DEGs identified by RNA sequencing in response to heat in potato are reliable.

### 2.8. Short and Prolonged Heat Stress Cause Metabolic Alterations in Potato Leaf

Potato leaves contain a large number of soluble metabolites, and most have not yet been identified. The nonbiased LC-MS global metabolomics approach detected up to 325 and 219 compounds in the positive and negative ionization modes, respectively, and they were up- or downregulated in leaves. Unlike the DEG number detected by RNA sequencing, the number of metabolites identified by LC-MS after 3 days of heat exposure was higher than that after 6 h of heat treatment, indicating that potato leaves adapt to tolerate elevated heat stress by increasing the number of defensive metabolites (Table 1, Table S6). Since many compounds are still unknown, to obtain further information on discriminant metabolites, 4 compounds under 6 h of stress and 16 compounds under 3 days of heat stress were identified as differentially expressed metabolites compared to the control (Table 2). Short heat stress suppressed the levels of secondary metabolite biosynthesis, such as stevioside, and prolonged heat stress decreased the biosynthesis of amino acids, such as histidine, and plant hormones, such as jasmonic acid. Both short and prolonged heat stress caused the upregulation of precursors of flavone and flavonol biosynthesis (quercetin, apigenin) and amino acid biosynthesis (L-proline, tyrosine), which indicates the higher accumulation of these metabolites.

To find patterns linking the transcriptome and metabolome, KEGG (Kyoto Encyclopedia of Genes and Genomes) pathways correlations were calculated based on fold changes of metabolites and transcripts. At least one metabolite and more than 100 transcripts for the biosynthesis of secondary metabolites and biosynthesis of amino acids under short heat stress were closely matched. After sustained heat stress, several pathways, including fatty acid biosynthesis, fructose, and mannose metabolism, carbon fixation in photosynthetic organisms and plant hormone signal transduction, were also activated and merged together at the metabolic and transcriptomic levels, indicating global changes in metabolic levels induced by prolonged heat stress to adapt to an elevated temperature (Table S8).

## 3. Discussion

Elevated temperature has negative effects on plant growth and development, and plants have evolved adaptive mechanisms in response to heat stress. Previous gene expression studies have mainly focused on potato responses to short heat treatment, and the effects of sustained heat treatment on potato responses and associated differences are poorly understood. Here, we treated potato plants with heat for 6 h or 3 days to comprehensively review new transcriptome and metabolism changes that occur under short and prolonged heat stress.

### 3.1. Short Heat Stress Maintaining the Stomata Open and Continued Stress Affect Photosynthetic Parameters

Stomata play a critical role in supporting fluxes of water and carbon dioxide in the soil-plant-atmosphere-climate system, and they also drive both plant mass transport and energy exchange. Generally, stomata have been shown to open wide in warm and moist environments but tend to close at low temperatures [19]. The results of our study showed that stomata were open after short heat treatment, which probably could lead to greater water loss from their leaves while allowing the plant to maintain efficient photosynthesis. In agreement with previous data that sustained heat stress triggered stomatal closure and induced a decline in the photosynthetic parameters Fv/Fm, yield (I) and yield (II), which were related to the maximum photosynthetic potential of the leaves and the real-time quantum yields of PSI and PSII, respectively [20]. Stomata have also been shown to close at increased temperatures [21]. However, elevated temperatures of approximately 30 °C have no effect on photosynthesis or severely inhibit photosynthesis for some heat-susceptible or heat-tolerant potato cultivars [22]. Compared with the work of Hancock et al. [6], who found that the contents of photosynthetic pigments chlorophyll a and chlorophyll b significantly decreased by up to 20% after moderately elevated temperatures of 30 °C during the day and 20 °C at night for up to 5 weeks, our results on potato exposure to heat after 3 days show that leaf chlorophyll a and chlorophyll b increased slightly at higher temperatures, which is consistent with previous studies [2]. A gene enrichment analysis revealed the enhanced expression of transcripts associated with aspects of PSII polypeptide subunits and ferredoxin by short and continued heat treatment. Despite the slight increase in the levels of photosynthetic pigments under continued heat treatment, all genes encoding chlorophyll a/b binding protein-associated photosystem II were significant decreased.

### 3.2. Upregulated Protective Proteins Are Characterized in Response to Heat Stress

Heat-shock protein (HSP) synthesis is involved in the adaptive response to heat exposure. The potato genome encodes 20 HSP70 and 48 sHSP (sHSP, HSP70, HSP90, HSP100) families [23,24]. In our study, 35 out of 37 heat stress-related transcripts and heat shock proteins were induced by short heat, and among them, 24 heat shock proteins were upregulated by continued heat treatment. These HSPs included 21 smHSPs and 8 HSPs with molecular masses from 70 and 130 kDa. smHSP accumulation is known to participate in thermotolerance in plants and represents a major group of genes involved in the heat response in higher plants [25]. HSP17.6 and HSP101 proteins strongly accumulated in the shoots and microtubers of the heat-sensitive potato cultivar Désirée and the heat-tolerant potato cultivar Festival in response to heat exposure [26]. In the potato, smHSPs were synthesized and accumulated at 35 and 40 °C. A previous study showed that the expression of an 18 kDa protein was significantly overexpressed when potato leaves were treated at 35 °C for 1 h. HSPs with molecular masses from 70 and 130 kDa increased their expression after heat exposure at 40 °C for 2 h [26]. The introduction of the heat shock protein gene encoding HSP17.7 to potato enhanced the plant’s heat tolerance [27], and a potato HSC70 gene has been recently demonstrated to protect against elevated temperature in potato [28]. Therefore, we conclude that moderate amount of HSPs were synthesized after the first moment of heat exposure and the majority of HSPs were further induced to respond to prolonged heat when heat stress was sustained. The present work also showed that the two DnaJ heat shock proteins were decreased after 6 h of heat treatment and one was decreased after 3 days of heat treatment. DnsJ proteins, either alone or in combination with heat-shock protein 70, act as molecular chaperones and take part in various important cellular processes, including protein assembly/disassembly, folding/unfolding, and degradation [29]. Moreover, DnaJ proteins also act as extremely important regulators of cellular protein homeostasis under stress conditions [30]; therefore, these DnaJ proteins as a co-chaperone may have a functional association with HSPs accumulation under heat stress in potato.

### 3.3. General and Specific Responses to Heat-Stress in Potato

When comparing heat-treated transcriptomes in potato leaves during development among three cultivars, 1364 genes were differentially expressed in “Hezuo 88”, 2190 genes were deregulated in “Desirée” from Hancock et al.’s [6] research, and 2949 genes were deregulated in “Agria” from Hastilestari et al.’s [13] research. We found that the number of DEGs identified in the current study was lower than that previously reported by Hancock et al. [6] and Hastilestari et al. [13] because of different methods for gene detection and screening. In addition, we used the third leaf counted from the top of the plant, although the heat treatment probably does not cause a large number of temperature-associated changes in gene expression in these tissues. However, Hancock et al. [6] reported the use of whole-compound leaves, including secondary leaflets, for DEG identification. The research of Hastilestari et al. [13] did not indicate which leaves were excised. Another explanation is that heat treatments of different temperatures and durations were applied in these studies. Hancock et al. [6] reported short/middle-term heat responses for 48 h of treatment, Hastilestari et al. [13] applied heat for 10 days, and we employed a short (6 h) and continued (3-day) heat treatment.

Our transcriptomes presented a higher degree of overlap of differentially expressed genes with those reported by Hastilestari et al. [13] than those published by Hancock et al. [6], which is because a prolonged heat treatment was applied in both studies and cv. Agria is more sensitive to elevated temperature than cv. Desirée [31]. Thus, we presumed that these genes are mostly involved in an adaptive response to high temperature. Another study using a yeast-based functional screening method identified 95 potential candidate genes after 2 and 48 h of treatment in Desiree. We found that 10 out of those 95 genes also showed increased expression in the current RNA-seq data. A comparison between our RNA-Seq data and previous microarray data showed that only 16 genes were commonly expressed genes; moreover, 370 upregulated and 837 downregulated genes were specifically identified by RNA-seq methods, indicating a number of new candidate genes that had not been previously linked to heat response. These commonly expressed genes attracted our attention since they are mainly involved in adaptive responses to heat and the production of heat shock proteins. Three class I heat shock protein genes and one nonspecific lipid-transfer protein gene were found among these studies, and they might act as molecular markers for assessing heat tolerance in potato during the development process. Indeed, the transient expression of these four genes in *Nicotiana benthamiana* resulted in greater membrane stability to heat treatment than controls.

### 3.4. Secondary Metabolism and Amino Acid Metabolism Involved in Heat Stress

The accumulation of secondary metabolites, such as flavonoids and phenylpropanoids, is correlated with the heat stress response and tolerance [32]. Secondary metabolites are involved in resistance against heat shock [33]. Short heat specifically induced the upregulation of isoprenoids, simple phenols, and their respective biosynthetic enzyme genes, and prolonged heat stress specifically induced the upregulation of phenylpropanoid genes. Sustained upregulation of six phenylpropanoid metabolism-related genes was found in potato leaves in response to heat stress (Figure S5). Phenylpropanoids are believed to play a critical role in the protection of plants against biotic and abiotic stress, and in many cases, these behaviours are associated with quenching reactive oxygen species after their synthesis [34]. The increase in the activity of phenylalanine ammonia-lyase (PAL), the key enzyme of the phenylpropanoid pathway, was reported as a major mechanism underlying the acclimation of cells to heat stress [35]. The precursor metabolites of flavone and flavonol biosynthesis accumulated in the potato leaves after heat treatment. The accumulation of various flavonols was also identified in a detailed metabolomics analysis of heat-stressed tomato [36]. The accumulation of anthocyanins, a class of flavonoids, was also induced by heat stress in vegetative tissues [37]. The downregulation of secondary metabolite biosynthesis, such as stevioside, after the start of heat stress exposure might be explained as a transient reaction to avoid adverse environmental conditions. The accumulation of some secondary metabolites under drought stress has also been reported in the potato [38]. Although few reports have considered the role of individual metabolites, we have demonstrated a role of these secondary metabolites in the response to heat stress at the transcriptomic and metabolic levels; moreover, we provide evidence that certain metabolites (flavone and flavonol) might play a specific protective role.

Various modifications in amino acid metabolism are closely related to abiotic stress in plants. Generally, the content of free amino acids in plants increases considerably during different abiotic stress conditions. In our experiment, 5 amino acid metabolism genes including glutamate decarboxylase, threonine, lysin, tyrosine degradation, and tryptophan synthesis were induced after 6 h heat exposure, 2 amino acid metabolism genes including aromatic amino acids and aspartate synthesis were consistently induced during experiment. In addition, the synthesis for proline and tyrosine were significantly upregulated on a metabolic level specifically at short and prolong heat stress, suggesting that these compounds are heat-responsive and putatively related to tolerance. Of these amino acids, proline is well-known, essential component for tolerating many environmental stresses in plants. The accumulation of proline is often recorded in response to a combined treatment of heat and drought stress [39]. Notably, the positive roles for proline in enhancing plant tolerance to heat remains controversial. In the first leaf of barley and radish, proline content was found to show a slight increase under 41 °C heat stress [40]. In contrast, proline accumulation was not observed during heat stress in *Arabidopsis thaliana* [41]. Exogenous application of proline can exaggerate the inhibitory effects of heat stress on seedling growth in *Arabidopsis thaliana* [41]. Expression of *AtP5CS1* gene in *Arabidopsis thaliana* confirmed that proline accumulation during heat stress is detrimental for plant growth [42]. Even so, it still appears that induction of proline is associated with the degree of leaf osmotic stress and the osmoregulation of genes involved in proline synthesis [43], indicating its role in osmoprotection.

## 4. Materials and Methods

### 4.1. Plant Material

Sprouted microtubers of the potato cultivar “Hezuo 88” from highland genotypes in China were planted in 1.0 L pot filled with vermiculite and cultivated in a glasshouse at 23 °C/21 °C for 16/8 h day/night periods, respectively. The light intensity was approximately 10,000 lx. After 8 weeks of cultivation, the plants with the same growth status were selected and transferred into a growth chamber for a 3-day adaption period with 14/10 day/night periods at 23 °C and then subjected to the heat treatment. For the high-temperature treatment, 24 plants were kept at 35 °C during light and at 33 °C during the dark phase, and samples were taken before the treatment and after 6 h and 3 days of stress application. The third fully unfolded leaves were collected, immediately frozen in liquid nitrogen, and stored at −80 °C until further assays.

### 4.2. Physiological Indices and Metabolic Assays

Potato plant leaves after 6 h and 3 days of heat exposure were detached and cut into strips 5 mm in length and 2 mm in width, followed by fixation with 4% glutaraldehyde at 4 °C for 48 h. The samples were subsequently washed three times with 0.1 M phosphate buffer for 10 min and then dehydrated in a graded series of ethanol (10, 30, 50, 70, 80, 90, and 95%) for 10 min each. After critical point drying, the samples were coated with gold for scanning electron microscopy (s-4800, Hitachi, Tokyo, Japan) analysis. Electrolyte leakage was measured according to previously described methods [44], and fresh leaf samples with a mass of approximately 100 mg were put into test tubes with 10 mL deionized water. The tubes were capped and placed in a water bath at 32 °C. The initial electrical conductivity (EC1) was recorded by an electrical conductivity meter (DDS-307A, Shanghai, China) after the samples were incubated at 32 °C for 2 h, and the final electrical conductivity (EC2) was measured for all released electrolytes after the samples were autoclaved at 121 °C for 20 min. The electrolyte leakage (EL) was calculated according to the formula EL = EC1/EC2 × 100. Photosynthesis system I and II (PSI and PSII) parameters were measured using a FMS-2 (Hansatech Instruments, England). The leaf chlorophyll was extracted with 12 mL *N*,*N*-dimethylformamide (DMF). After 72 h of extraction at 4 °C in the dark, the absorbance of the supernatant solution at 647 nm and 664.5 nm was measured with a spectrophotometer, and the concentration of total chlorophyll was calculated according to the equations of Inskeep and Bloom [45]. Chlorophyll was calculated as ug·g^−1^ of tuber surface. For both the control and stress treatments, three biological replicates were performed for these physiological index measurements.

### 4.3. RNA Samples Preparation and High-Throughput Sequencing

Total RNA was isolated using TRIzol Reagent (Tiangen, Beijing, China) following the manufacturer s’ instructions. The RNA integrity and concentration were measured by electrophoresis on 1.0% agarose gels and a NanoPhotometer^®^ spectrophotometer. Paired-end sequencing libraries with average insert sizes of 300–400 bp were generated with AMPure XP beads and sequenced on a HiSeq 4000 (Illumina, San Diego, USA) following the manufacturers’ guidelines. Raw data were processed and filtered by the Illumina pipeline to generate FastQ files (http://www.Illumina.com). After that, approximately 150-bp clean reads were obtained from 6 sequenced libraries. The genome sequence of potato retrieved from the Potato Genomics Resource (PGSC) acts as the reference. All the clean reads were then mapped onto the PGSC by STAR (v2.5.1b). The number of reads mapped to each gene was counted using HTSeq v0.6.0, and then the abundance of transcripts were normalized by FPKM (fragments per kilobase per million). The gene abundance difference of two treatments with one biological replicate each was determined using the negative binomial distribution model with the DESeq2 R package (1.10.1), and the false discovery rate (FDR) was calculated by adjusting the *p*-values using Benjamini and Hochberg’s approach.

### 4.4. Metabolite Extraction, Measurement and Analysis

One hundred milligrams of powdered leaf material were extracted with 400 µL of 80% methanol at −20 °C for 1 h. The solution was centrifuged at 14,000× *g* for 20 min at 4 °C, and the extraction solvent was removed in a Speed-Vac. The dried samples were resolved in 100 µL of 80% methanol in water and centrifuged at 14,000× *g* for 15 min at 4 °C. LC-MS measurements were performed on a QE HF-X coupled to a Vanquish UHPLC (Thermo Fisher Scientific Inc, Massachusetts, CA, USA). Chromatographic separation was achieved on an Accucore HILIC column. Eluent A was 95% acetonitrile in water with 0.1% formic acid, and eluent B was 50% acetonitrile with 0.1% formic acid. The flow rate was 300 mL·min^−1^, and the column temperature was set at 40 °C. Mass calibration was achieved with low-concentration ESI Tuning Mix (Agilent Technologies, Santa Clara, CA, USA). The mass spectrometer was operated as follows: spray voltage: 3.2 kV; sheath gas flow rate: 35 arb; aux gas flow rate: 10 arb; capillary temperature: 320 °C; and polarity: positive/negative. Mass spectra were acquired in a mass range of 100 to 1500 *m*/*z*. The raw data files were separately imported into Compound Discoverer 3.0 to carry out peak detection. The resulting peaks were normalized and used for molecular formula prediction. The molecular formula was further imported into the mzCloud and ChemSpider databases for accurate relative quantification. The discriminant metabolites were determined by OPLS-DA.

### 4.5. Transient Expression in Nicotiana benthamiana

Plasmid pBi121 containing target sequences was transferred to *Agrobacterium* strain LBA4404. *Agrobacterium*-mediated transformation of *Nicotiana benthamiana* was conducted as previously described [28]. Leaves of *Nicotiana benthamiana* plants (growing conditions: 23 °C day and 21 °C night in a 16 h-light/8 h-dark cycle) were agroinfiltrated by a needleless syringe. After 12 h of incubation at 22 °C, the agroinfiltrated plants were transferred to a growth chamber at 45 °C with 12 h photoperiod under a light intensity of 10,000 lx. After 24 h, the leaves were harvested for electrolyte leakage measurement as mentioned above.

### 4.6. Real-Time qRT–PCR Analysis

First-strand cDNA was synthesized from 0.5 μg of total RNA, as mentioned above, using the Prime ScriptTMRT reagent Kit with gDNA Eraser (Takara, Dalian, China) according to the manufacturers’ instructions. Gene-specific primers were designed based on the corresponding potato gene DNA sequences using Primer 3 (version 0.4.0) (Table S8). Each reaction included 5 μL of 2×Plus SYBR real-time PCR mixture (BioTeke, Beijing, China), 1 μL of cDNA, 0.5 μL of each forward and reverse primer, and 3 μL of sterile H_2_O following the manufacturer’s protocol. Three biological replicates with three technical replicates were subjected to qRT-PCR measurement using CFX96TM Real-Time System (BIO-RAD, Hercules, CA, USA) for each sample. Fold change was calculated using the $2^{-\Delta \Delta CT}$ method in comparison with ubi3 as an internal control gene [46].

## 5. Conclusions

In summary, the short and prolonged heat treatment of potato leaves resulted in lower membrane stability and decreased photosynthetic parameters, which were accompanied by transcriptomic and metabolomic changes. DEGs enriched in photosynthesis, cell wall degradation, response to heat, RNA processing, and protein degradation were highly induced during heat exposure, and differentially expressed metabolites involved in amino acid biosynthesis and secondary metabolism were mostly induced during heat exposure, suggesting a possible role of these genes and metabolites in the heat tolerance of the potato. Heat stress response genes overlap and are conserved with drought stress response genes in potato and heat stress response genes in *Arabidopsis* shoots. These datasets provide evidence that transcriptional and metabolic changes are adaptive responses to short and prolonged heat exposure in potato plants.

## Figures and Tables

**Table 1:** Number of differentially expressed genes and metabolites in response to 6 h and 3 days heat stress.

	H6h	Overlap	H3d
Transcriptome			
DEG up	160	157	130
DEG down	538	285	94
TF up	14	6	7
TF down	56	30	14
Metabolome (−)LC-MS			
Compound up	24	12	86
Compound down	15	10	94
Metabolome (+)LC-MS			
Compound up	10	3	115
Compound down	44	32	156

DEG = differentially expressed gene; TF = transcription factors; H6h, potato leaves after 6 h heat exposure; H3d, potato leaves after 3 days heat exposure; Compound is defined by the sum of the molecular weights of its elements.

**Table 2:** Important metabolites involved in heat stress identified by partial least square discriminant analysis and significant analysis of metabolites.

ID	Formula	Kegg_ID	Pathway Name	log_2_FC	*p* Value	VIP
H6h vs. HC						
Com_99_neg	C38H60O18	cpd:C09189	Biosynthesis of secondary metabolites	−1.56891	0.032739	1.946182
Com_224_neg	C26H28O16	cpd:C12637	Flavone and flavonol biosynthesis	1.188334	0.013747	1.470693
Com_232_neg	C8H9NO3	cpd:C00250	Metabolic pathways	1.297375	0.032787	1.604524
Com_608_neg	C5H9NO2	cpd:C00148	Biosynthesis of amino acids	2.598589	0.00482	3.211703
H3d vs. HC						
Com_330_neg	C16H30O2	cpd:C08362	Fatty acid biosynthesis	−3.80026	0.000541	2.634698
Com_1053_neg	C27H44O3	cpd:C01673	Steroid biosynthesis	−3.62372	0.002722	2.513606
Com_22_neg	C8H10O2	cpd:C06044	Tyrosine metabolism	2.141449	0.024333	1.481691
Com_605_neg	C28H44N2O8S	cpd:C06462	Arachidonic acid metabolism	1.843223	0.005207	1.278471
Com_224_neg	C26H28O16	cpd:C12637	Flavone and flavonol biosynthesis	1.770409	0.025374	1.228481
Com_1186_neg	C26H28O14	cpd:C04858	Flavone and flavonol biosynthesis	1.644199	0.031581	1.137737
Com_1450_pos	C3H7O6P	cpd:C00118	Glycolysis/Gluconeogenesis	−1.7076	0.033928	1.322058
Com_1193_pos	C6H9N3O2	cpd:C00135	Biosynthesis of amino acids	−1.48884	0.021547	1.149797
Com_566_pos	C2H5O4P	cpd:C03167	Phosphonate and phosphinate metabolism	−2.13038	0.002935	1.646439
Com_2095_pos	C30H54N10O10S2	cpd:C16564	Glutathione metabolism	−1.92443	0.010559	1.488118
Com_3261_pos	C18H32O4	cpd:C04717	Linoleic acid metabolism	−3.14728	0.000754	2.432073
Com_1277_pos	C12H18O3	cpd:C08491	Plant hormone signal transduction	−1.32988	0.003432	1.028105
Com_1814_pos	C24H29NO10	cpd:C11813	Isoquinoline alkaloid biosynthesis	−1.42629	0.009259	1.101574
Com_1804_pos	C21H30O3	cpd:C03205	Metabolic pathways	1.348268	0.046435	1.038995
Com_150_pos	C7H8	cpd:C01455	Metabolic pathways	3.252694	0.037825	2.494752
Com_517_pos	C21H30O2	cpd:C00410	Metabolic pathways	1.928316	0.035943	1.501117

VIP = variable importance in projection.

## Data Availability

The RNA sequence data generated in this study have been deposited at NCBI in the Short Read Archive database under the BioProject accession number PRJNA588378.

## Supplementary Materials

The following are available online at https://www.mdpi.com/2223-7747/10/1/103/s1, Figure S1. MapMan display of all present genes. Gene expression is illustrated in the metabolism overview (A, C, E) and the cellular response overview (B, D, F) after 6 h and 3 days of heat stress, respectively. Color bar ranging from −2.0 to +2.0 ($\log_{2}value$). Color patterns from blue to red indicate the expression levels of induced and reduced gene expression in heat-stressed samples versus control samples. Genes with no significant change in the amplitude are shown in white. Figure S2. Transcription factors genes upregulated and downregulated after heat exposure at 6 h and 3 days. A. Specially upregulated TFs after 6 h heat stress; B. Specially downregulated TFs after 6 h heat stress; C. Specially upregulated TFs after 3 days of heat stress; D. Specially downregulated TFs after 3 days heat stress; E. Commonly upregulated TFs after 6 h and 3 days of heat stress; F. Commonly downregulated TFs after 6 h and 3 days heat stress. Figure S3 Transient expression of heat induced genes in *Nicotiana benthamiana* leaves. A. Phenotype of transgenic *Nicotiana benthamiana* plants after 24 h at 45 °C. B. Membrane damage in agro-infiltrated *Nicotiana benthamiana* plants after one day at 45 °C by electrolyte leakage assay compared with agro-infiltrated plants with empty vector (Mock). Asterisks indicate significant difference between treatments as estimated using Student’s *t*-test (*p* < 0.05). Error bars represent the standard error of the mean (*n* = 3). Figure S4 Analysis of mRNA level by RT–qPCR. The mRNA level of 20 randomly selected genes was analysed by RT–qPCR. The average of results obtained from three plants is represented (left Y-axis) together with the RNAseq data (right Y-axis), data are represented as mean ± SE (*n* = 3). Figure S5. Secondary metabolism overview in MapMan. A. Specifically upregulated and downregulated genes after 6 h heat exposure; B. Specifically upregulated and downregulated genes after 3 days heat exposure; C. Commonly upregulated and downregulated genes after 6 h and 3 days heat stress. Table S1. Number of up- and downregulated genes after 6 h and 3 days heat stress. Table S2. Number of up- and downregulated transcription factors after 6 h and 3 days heat stress. Table S3. Results of the comparative analysis of heat-responsive genes in potato leaves and *Arabidopsis thaliana* shoots. Table S4. Specifically and commonly expressed genes in heat-stressed potato and drought-stressed potato. Note that drought-responsive transcripts were extracted from greenhouse trials. Table S5. Number of common elements in the heat stress and published datasets. Table S6. Significantly accumulated metabolites with their compound ID and molecular formula, identified by partial least square discriminant analysis from short and prolonged heat stressed potato leaves. Table S7. Mapping of transcripts onto metabolite data using KEGG. Table S8. Primer list used in qPCR analysis.
