# Supplementary figures and images for: Gene and Metabolite Integration Analysis through Transcriptome and Metabolome Brings New Insight into Heat Stress Tolerance in Potato (Solanum tuberosum L.)
Source: Plants (Basel). 2021 Jan 6;10(1):103. doi: 10.3390/plants10010103 (PMC7825342; doi:10.3390/plants10010103)

Figure S1

**A**


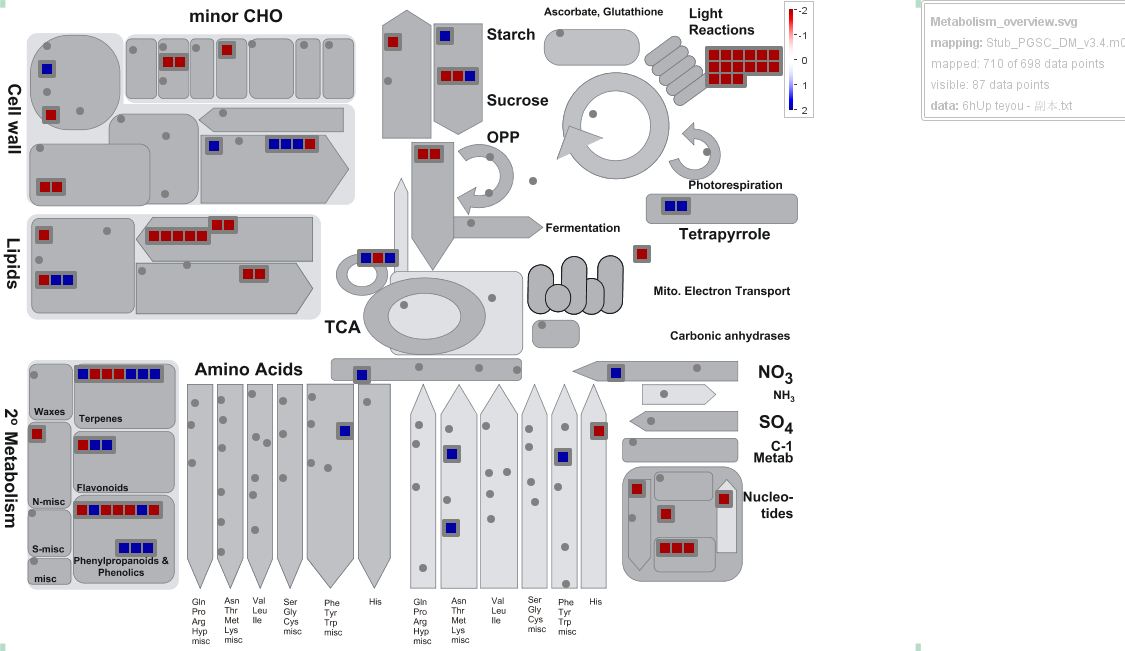

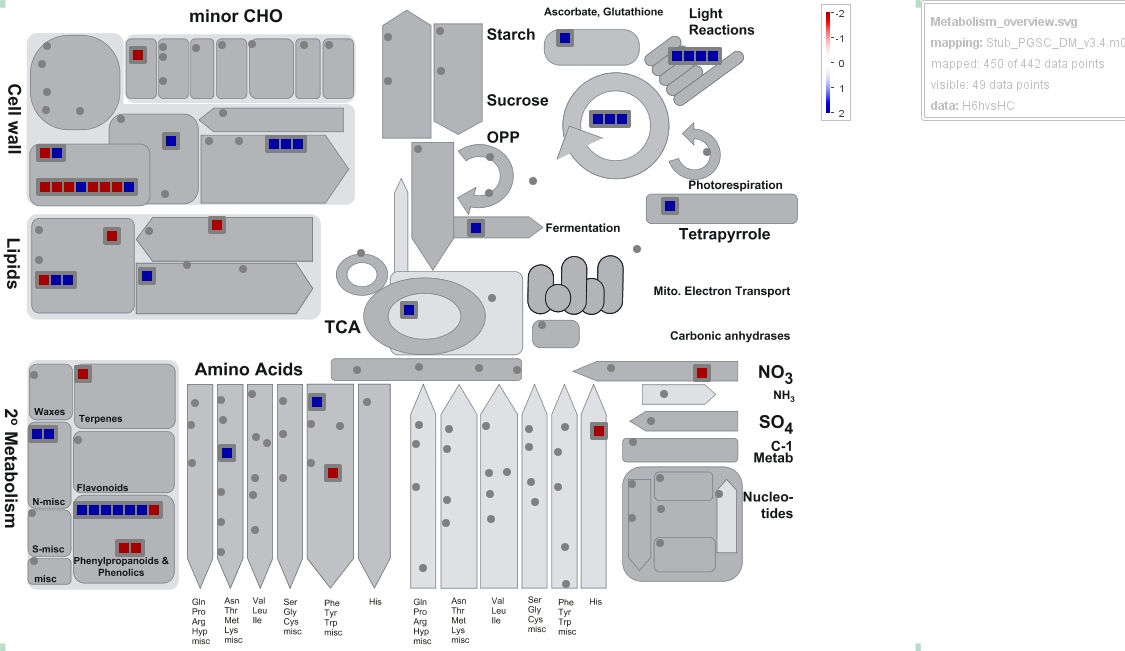


**C**


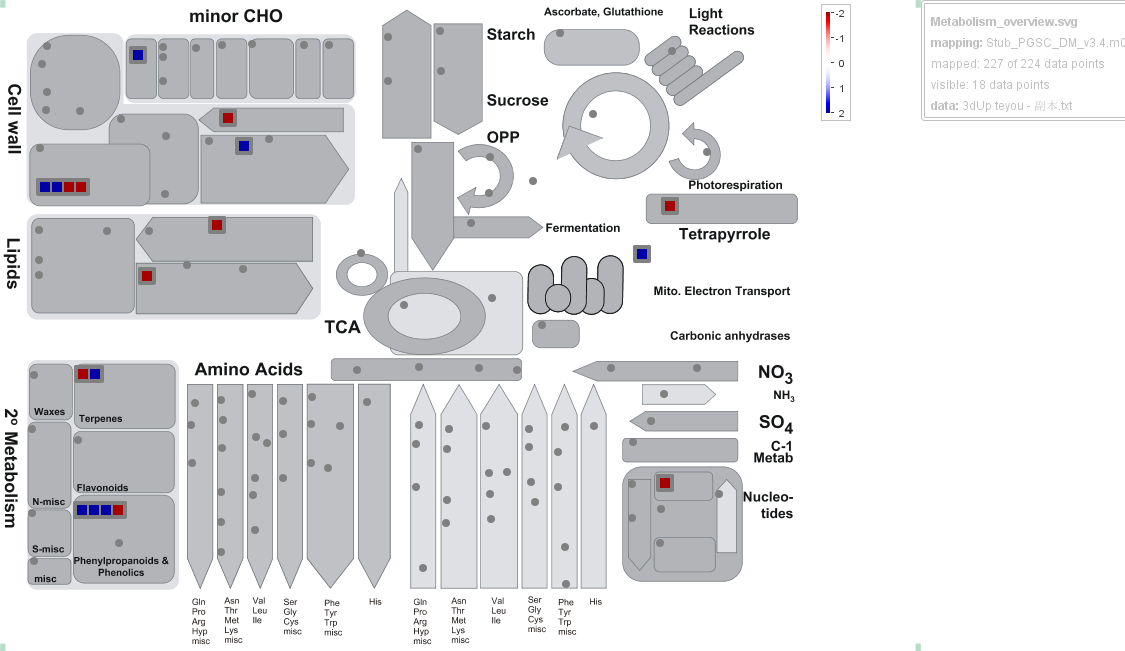


**E**


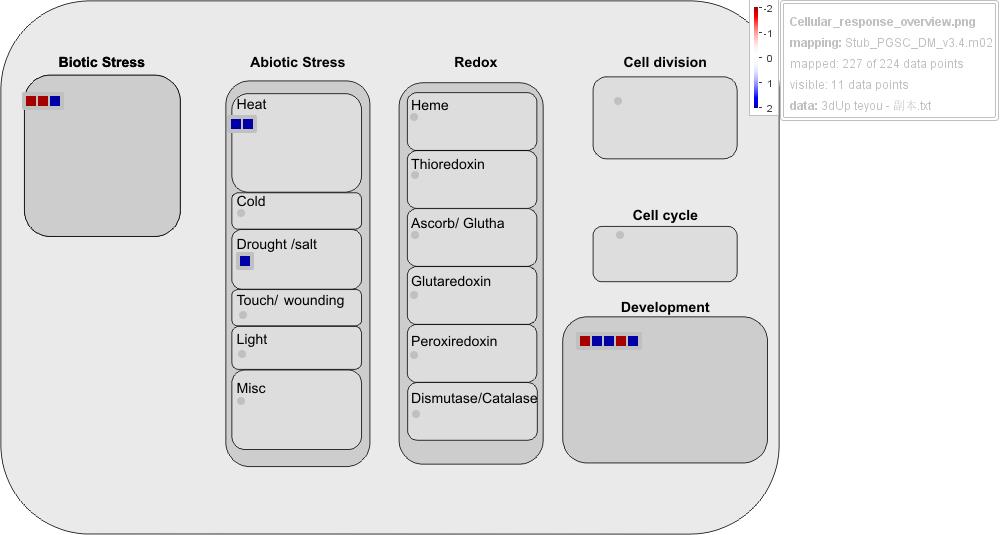

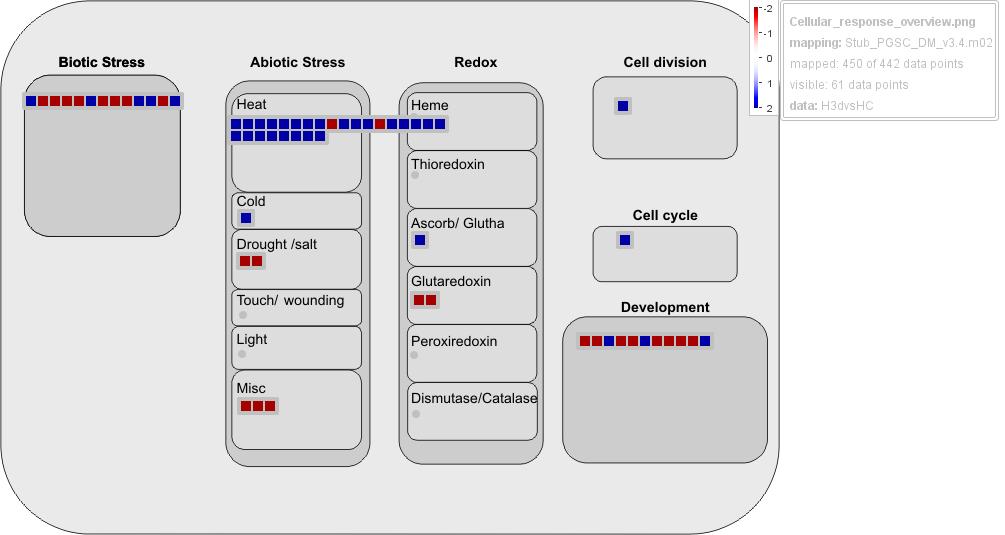


**F**

**D**


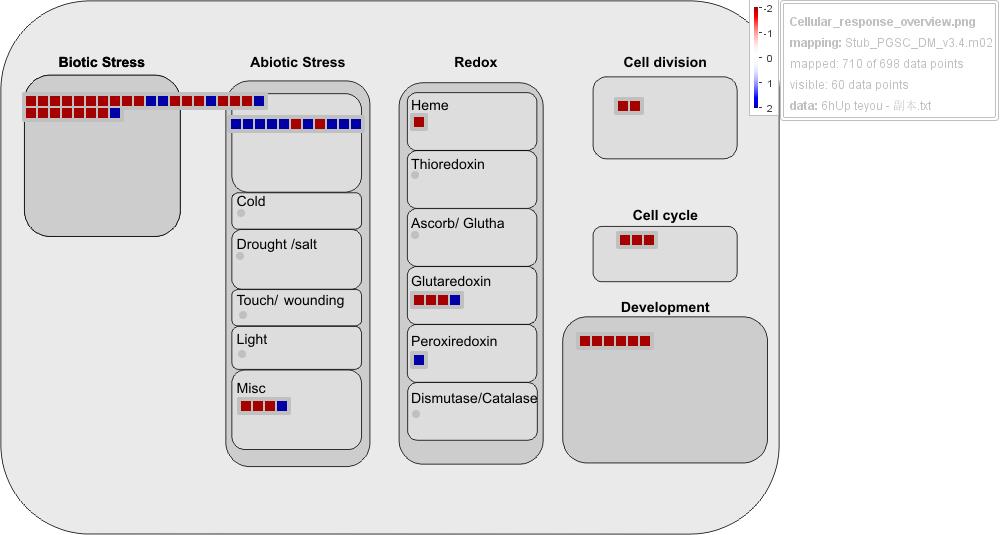


Figure S2


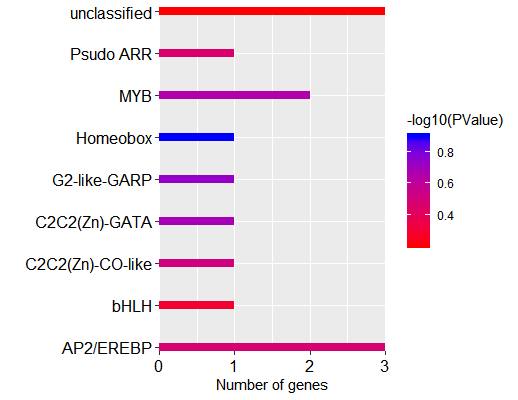

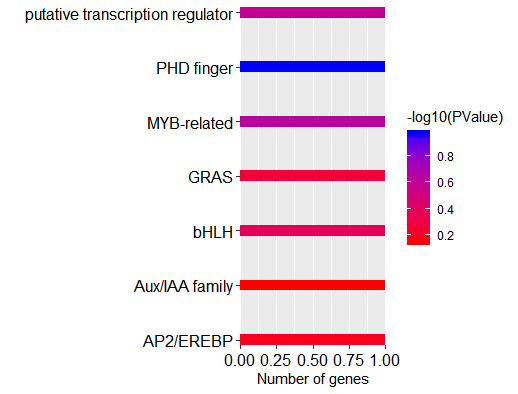

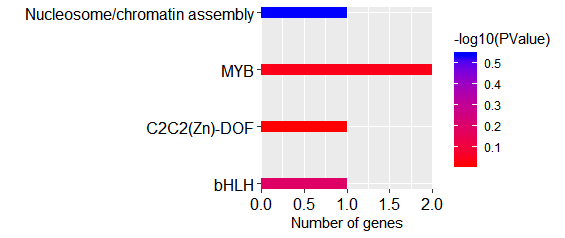

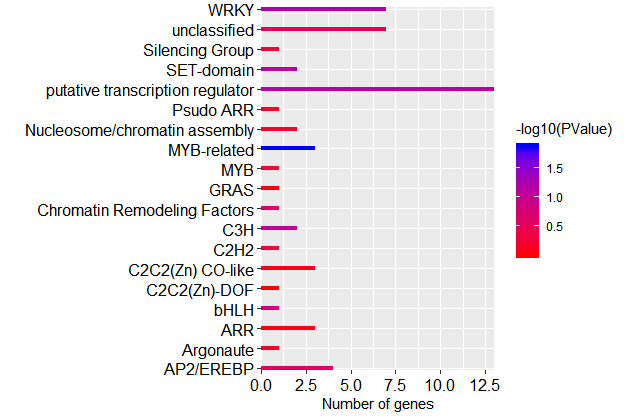

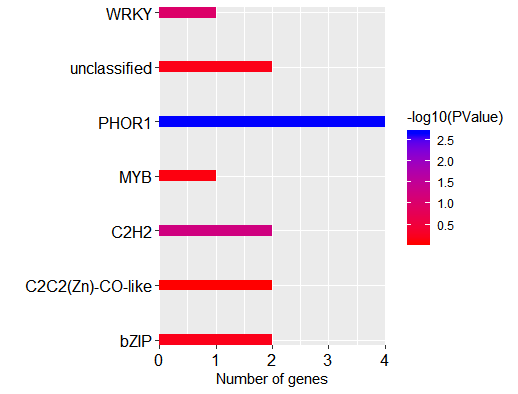

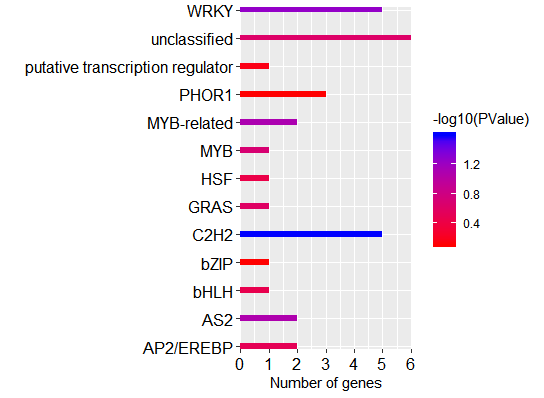


A

B

C

D

E

F

Figure S3


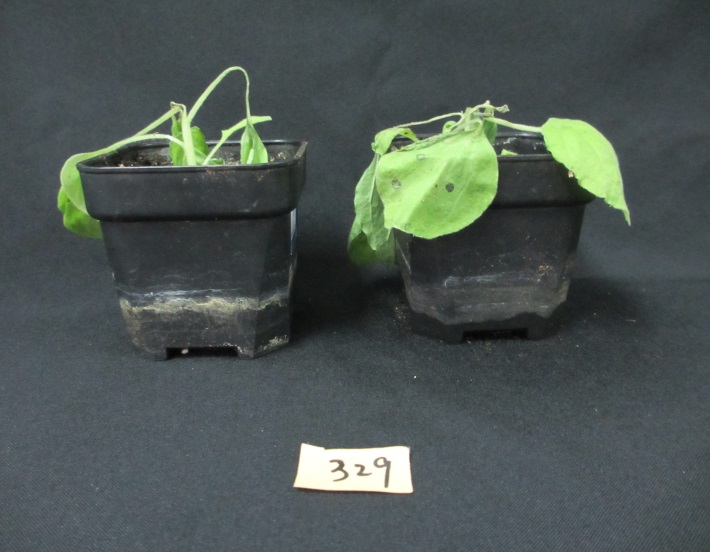

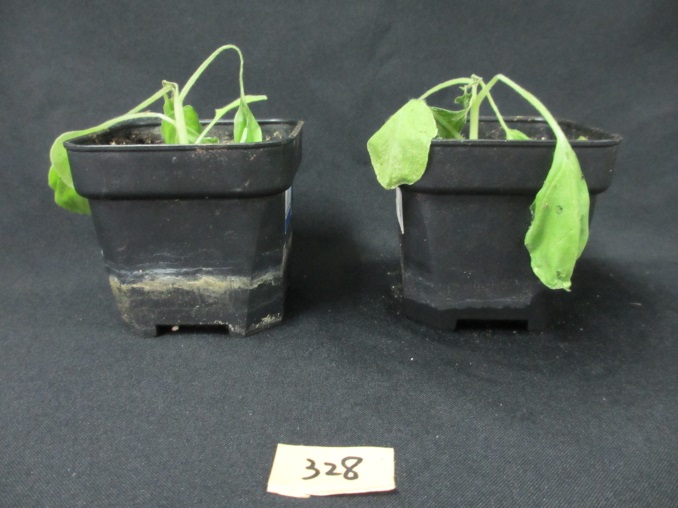

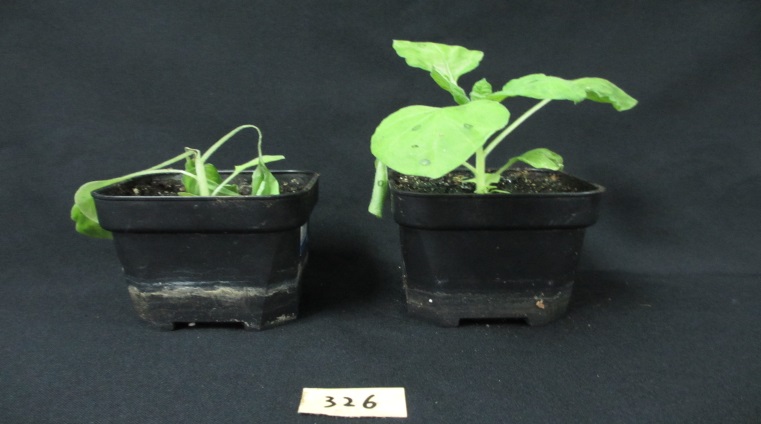

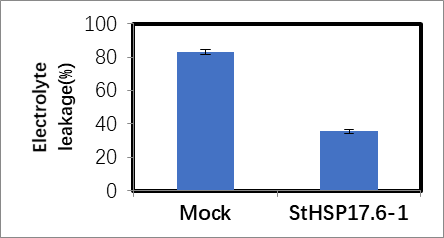

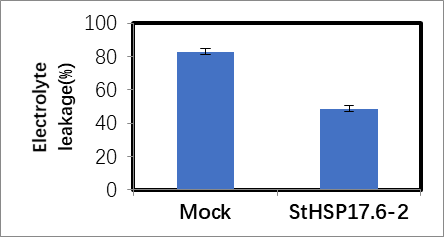

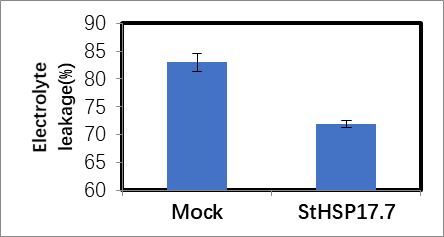

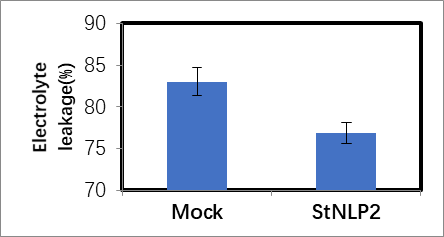


*

*

*

*


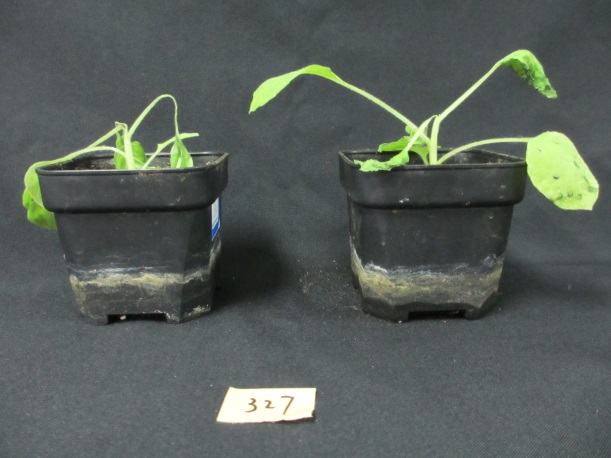


**A**

**B**

**Mock**

**Selected genes**

Figure S4


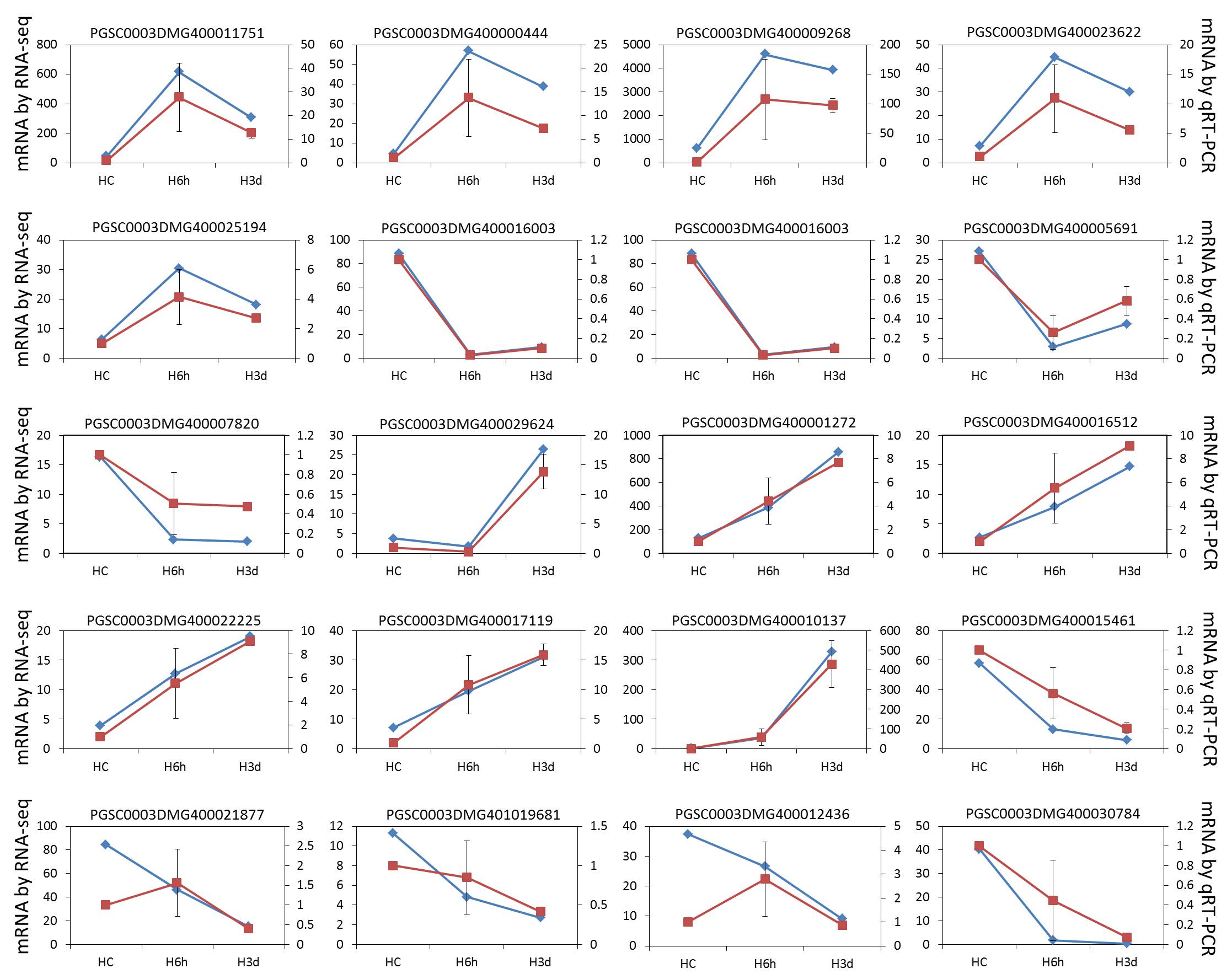


Figure S5


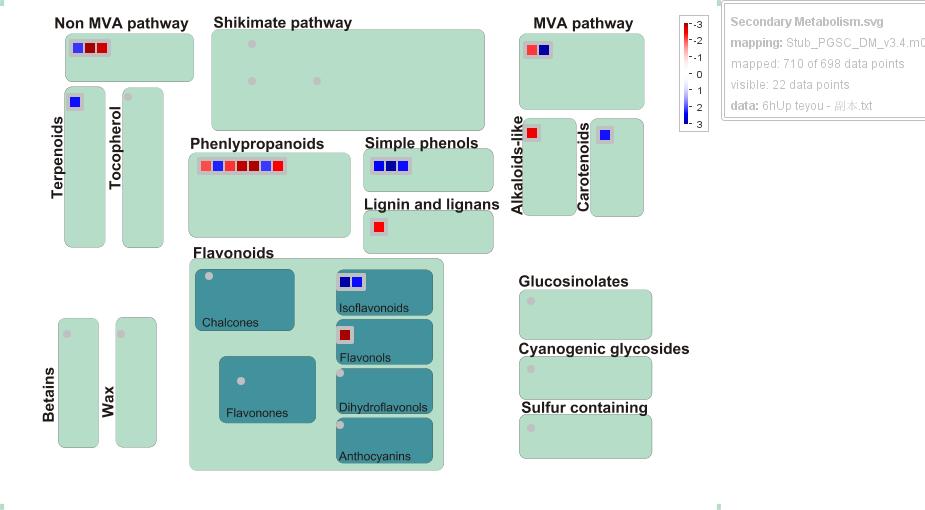


**A**


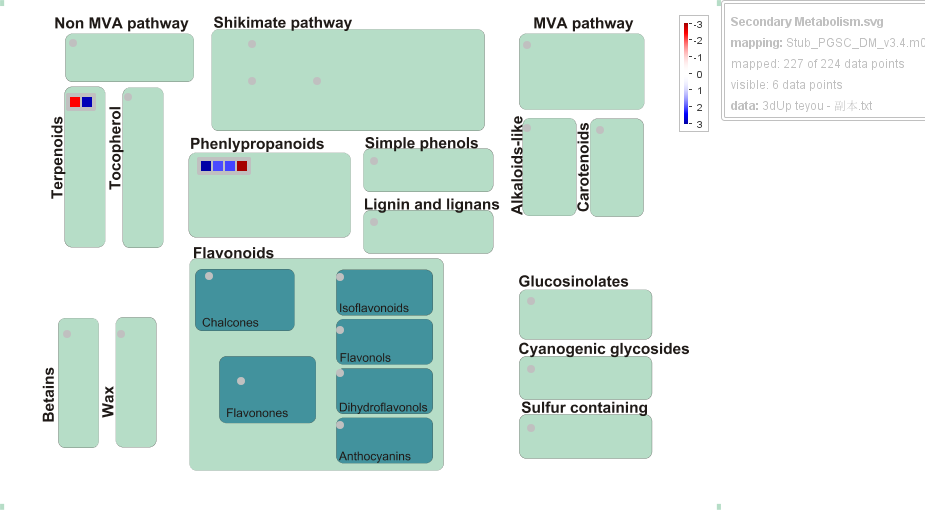


**B**


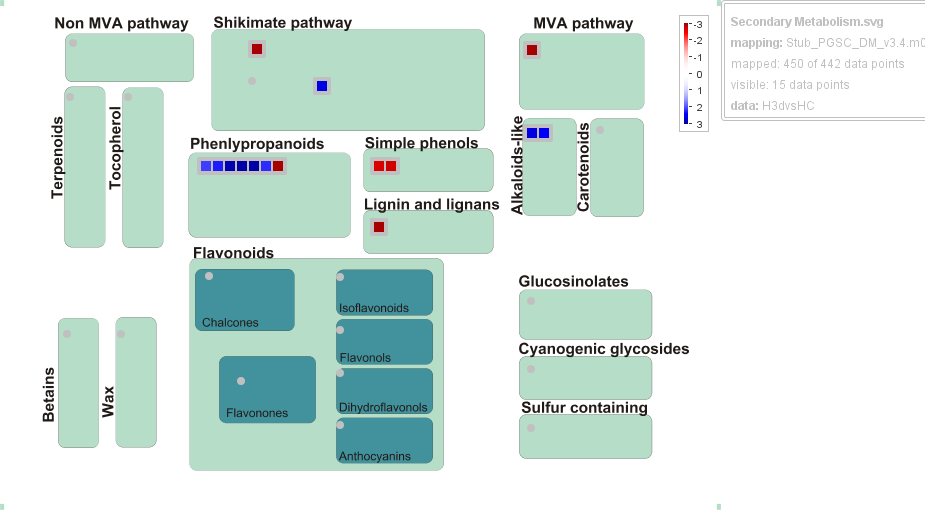


**C**

Supplement: Supplementary file 1 [file plants-10-00103-s001.zip › FigureS1-S5.docx]
